# Simulation-based inference of cell migration dynamics in complex spatial environments

**DOI:** 10.1038/s41540-026-00648-9

**Published:** 2026-01-29

**Authors:** Jonas Arruda, Emad Alamoudi, Robert Mueller, Marc Vaisband, Ronja Molkenbur, Jack Merrin, Eva Kiermaier, Jan Hasenauer

**Affiliations:** 1 Life & Medical Sciences Institute, University of Bonn, Bonn, Germany; 2 Bonn Center for Mathematical Life Sciences, University of Bonn, Bonn, Germany; 3 Department Information Services and High Performance Computing, Center for Interdisciplinary Digital Sciences, TUD Dresden University of Technology, Dresden, Germany; 4 Life & Medical Sciences Institute, Immune and Tumor Biology, University of Bonn, Bonn, Germany; 5 Institute of Science and Technology Austria, Klosterneuburg, Austria; 6 Department of Medicine 1/CITABLE, Friedrich-Alexander University of Erlangen-Nürnberg, Erlangen, Germany; 7 Deutsches Zentrum Immuntherapie (DZI), Universitätsklinikum Erlangen, Erlangen, Germany

**Keywords:** Biophysics, Computational biology and bioinformatics, Mathematics and computing, Physics, Systems biology

## Abstract

To assess cell migration in complex spatial environments, microfabricated chips, such as mazes and pillar forests, are routinely used to impose spatial and mechanical constraints, and cell trajectories are followed within these structures by advanced imaging techniques. In systems mechanobiology, computational models serve as essential tools to uncover how physical geometry influences intracellular dynamics; however, decoding such complex behaviors requires advanced inference techniques. Here, we integrated experimental observations of dendritic cell migration in a geometrically constrained microenvironment into a Cellular Potts model. We demonstrated that these spatial constraints modulate the motility dynamics, including speed and directional changes. We show that classical summary statistics, such as mean squared displacement and turning angle distributions, can resolve key mechanistic features but fail to extract richer spatiotemporal patterns, limiting accurate parameter inference. To solve this, we applied neural posterior estimation with in-the-loop learning of summary features. This learned summary representation of the data enables robust and flexible parameter inference, providing a data-driven framework for model calibration and advancing quantitative analysis of cell migration in structured microenvironments.

## Introduction

Tissues are complex, highly organized systems comprising cells and extracellular components that exhibit dynamic behavior and structural heterogeneity across developmental stages. This spatiotemporal complexity, encompassing both biochemical gradients and mechanical cues, critically influences cell behavior, particularly migration dynamics within structured or constrained environments^1,2^.

Coordinated migration of immune cells is a prerequisite for a properly operating immune system. One prominent route of immune cells is the migration of dendritic cells (DCs) from the periphery to secondary lymphoid organs such as lymph nodes by following chemokine gradients^3^. Such guided migration, termed chemotaxis for soluble attractants and haptotaxis for immobilized chemokine gradients, arises from chemokine-receptor engagement and downstream signaling that coordinate chemotactic sensitivity, migration persistence, cell morphological dynamics, and traction forces within the chemically and mechanically defined microenvironment^4^.

Advances in live cell imaging have facilitated the acquisition of extensive quantitative data to capture the migration of cells^5^. The in-depth interpretation of these data necessitates computational models, which capture multiscale interactions and describe emergent behaviors. State-of-the-art modeling approaches, including discrete and continuous models in both temporal and spatial domains, as well as hybrid frameworks, have been employed to capture cell behavior and cell-cell interactions within complex environments^6–10^. Specifically, Cellular Potts models^11^ and vertex models, which are continuous in time and discrete in space, are utilized to simulate cell interactions, with numerous software platforms available for implementing and testing these models^12–17^. Unlike stochastic differential equation approaches, which primarily capture the motion of cell centroids, the Cellular Potts model explicitly represents spatially extended cells, allowing direct modeling of shape deformations and interactions between cells and their environment. This makes it particularly suitable for studying migration dynamics in structured environments where spatial effects dominate. Various studies have leveraged such simulations to unravel the dynamics of chemotaxis and cell migration, emphasizing the pivotal role of computational models in understanding cellular behavior^18,19^^,20,21^.

A central challenge in applying these models is their calibration using experimental data. Typically, model parameters are not directly measurable, yet they are crucial for elucidating biological mechanisms. Parameter estimation enables quantitative modeling^22,23^, hypothesis testing^24^, and prediction of dynamic responses to perturbations^25,26^. The inference of cell migration-related parameters has been previously explored^27,28^. State-of-the-art approximate Bayesian computation (ABC) sequential Monte Carlo pipelines facilitate model calibration based on sequential rounds of simulations updating the posterior without the need to derive a likelihood, but rely on summary statistics to reduce the dimensionality of the data^29–31^. These statistics, such as the mean squared displacement or turning angle distributions of cell movement, are often hand-crafted based on domain knowledge. However, they may fail to provide relevant information for precise parameter estimation. To address these challenges in the context of ABC, recent approaches have included adaptive algorithms that weight hand-crafted summaries to maximize the posterior information gained from a dataset or to learn summaries on pairs of parameters and simulations^32–34^. However, hand-crafted summaries or summaries based on the posterior mean may not be sufficient to reconstruct the posterior shape^34,35^. As ABC methods require a large number of model simulations, their applicability in the context of complex migration models remains unclear.

Alternatives to ABC, such as neural posterior estimation (NPE) methods, employ neural networks to learn mappings from data to model parameters, effectively handling high-dimensional data and sophisticated models and circumventing the limitations of conventional ABC methods^36^. By training neural networks, relevant features can be extracted automatically from the data, and NPE can improve parameter estimation accuracy^37^. Extensive studies have shown the successful application of NPE on various datasets^38,39^, but not yet in the context of cell migration in spatial environments.

In this manuscript, we assessed the application of classical and machine learning simulation-based inference methods for the analysis of cell migration in complex environments. We implemented a comprehensive inference pipeline to evaluate the different inference approaches. As an important example of the interplay between spatial structure and cell migration, we focused on DC movement in complex environments. Therefore, we designed microstructured polydimethylsiloxane chips that impose obstacles on migrating DCs and recorded their trajectory data. To analyze the data, a Cellular Potts model of migration in a spatially complex environment was constructed, and ABC with different summary statistics was compared with NPE using simulated and experimental data.

Through simulation studies, we demonstrated that the model captures the effects of spatial confinement on cell migration, highlighting the importance of incorporating complex geometries in quantitative analyses of cell migration. Subsequently, we showed that the parameters used for the simulation study can be reconstructed using tailored inference methods. Following these results, we applied our inference pipeline to the experimental data to analyze how environmental mechanics and structure influence cell behavior.

## Results

To quantitatively dissect how chemokine gradients and physical constraints influence DC migration, we designed a structured in vitro assay and built a mechanistic in silico model based on the Cellular Potts framework. We performed parameter inference using likelihood-free methods, as the Cellular Potts model lacks an explicit likelihood function that would allow classical Bayesian methods, like Markov chain Monte Carlo. Using synthetic data with known ground truth, we systematically evaluated four simulation-based inference strategies: (i) approximate Bayesian computation sequential Monte Carlo (ABC) with hand-crafted summary statistics (normalized displacement, velocity, turning angle, and angle degree); (ii) ABC employing a neural network as a feature extractor trained either to predict the posterior mean (ABC-PM) or (iii) with summaries tailored for inference (ABC-NPE); and (iv) NPE with jointly trained summary networks (as described in the “Methods”). We then applied the pipeline to calibrate our model on experimentally tracked DC trajectories under chemotactic stimulation within a microstructured polydimethylsiloxane (PDMS) pillar array, offering mechanistic insights into how chemical signals and spatial barriers jointly govern immune cell navigation.

### Experimental analysis of DC migration in pillar forests reveals high cell-to-cell variability

**Fig. 1:**
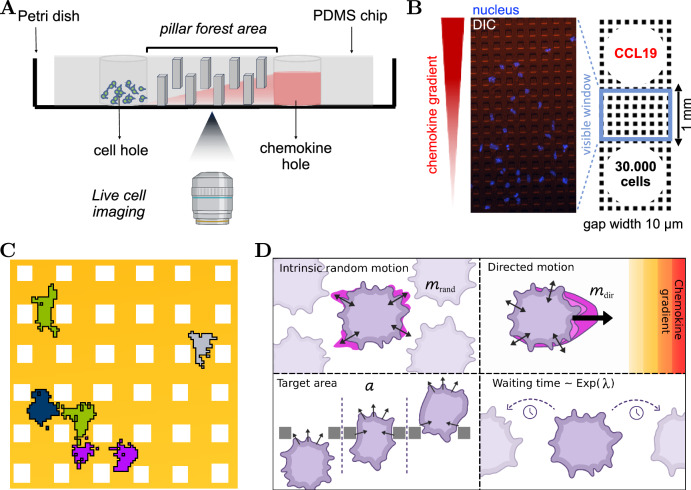
Schematic depiction of the experimental and modeling setup. **A** Dendritic cells were placed in an entry hole on the left and followed a chemokine gradient to the right through the pillar forest on a microstructured polydimethylsiloxane (PDMS) chip. **B** Top-down differential interference contrast (DIC) microscopy view within the visible window of the pillar forest, where cells were tracked within the first 2 h of imaging. Dextran was added to the CCL19 solution to visualize the chemokine gradient. **C** Zoomed-in visualization of a simulation of the Cellular Potts model (CPM). **D** Visualization of the four model parameters to be estimated in the CPM: the intrinsic random motion strength *m*_rand_ (upper left tile), the chemokine attraction strength *m*_dir_ (upper right tile), the target area of a cell *a* (lower left tile), and the rate *λ* until the cell changes its intrinsic direction (lower right tile). Parts of this figure were created using BioRender.

DCs must efficiently migrate through complex tissue environments to reach lymphoid organs, guided by chemokine gradients. Understanding how they integrate chemical signals with physical constraints is critical for deciphering the mechanisms of the immune system. To study this interplay, we exposed bone marrow-derived DCs to a stable CCL19 gradient and confined them within a microfluidic chip containing a pillar forest with 10 μm gaps (Fig. 1A and ref. ^40^). Time-lapse videomicroscopy was used to track the nuclear positions at 30-s intervals in the visible window (Fig. 1B), yielding trajectories of variable length depending on how long a cell remained within the field of view (further experimental details in “Methods”).

The analysis of the tracks revealed that cells follow the chemokine gradient, whose strength decreases with distance from the chemokine hole (Fig. 1B and ref. ^41^). Yet, we observed a high cell-to-cell variability. Indeed, only a subset of 147 cells migrated toward the chemokine source within the first 2 h of imaging, while some did not respond to the gradient, moved outside the field of view, or shifted along the *z*-axis. For a quantitative assessment comprehensive of the process, we assess four descriptive measures of migration (see “Methods”): (1) Normalized displacement, which quantifies overall progress across the track; (2) Velocity, which reflects the average speed of migration; and (3) Turning angle and (4) Angle degree distributions, which characterize directional biases toward the chemokine gradient or deflections caused by obstacles.

These summary statistics formed the basis for quantitative comparison with a mechanistic model, linking experimental single-cell measurements to simulations and providing insight into how DCs balance chemokine guidance with geometric restrictions imposed by tissue-like barriers.

### Integrative modeling and inference pipeline enables simulation and inference

To analyze DC migration, we developed a 2D Cellular Potts model with chemotactic signaling and persistent random motion embedded in a simulation-based inference pipeline. In this lattice-based stochastic framework, cells are represented as extended domains of lattice sites, allowing the simulation of realistic cell shapes and their deformations. Cell behavior emerges from an energy minimization principle that combines terms for volume constraints with responses to external cues, such as chemokine gradients, allowing us to generate trajectories under conditions that mimic the experimental setup (Fig. 1C). Model simulations provide cell trajectories and allow for the computation of the afore-described summary statistics.

The model possesses four parameters with a substantial influence on the migration patterns (Fig. 1D): the chemokine attraction strength *m*_dir_, which quantifies the sensitivity of cells to the chemokine gradient modulating directed movement; the intrinsic random motion strength *m*_rand_, which governs the bias toward previously randomly chosen movement directions; the rate *λ* for the waiting-time distribution between directional changes of the intrinsic random motion; and the target area *a*, which regulates cell size via area constraints (for details, see “Methods”).

**Fig. 2:**
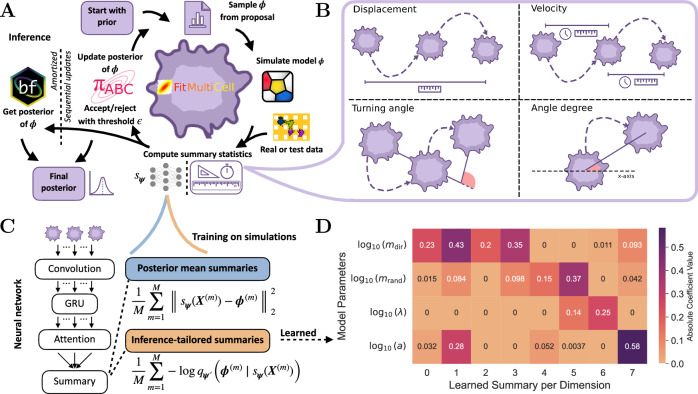
Overview of our pipeline for parameter inference in cell migration models. **A** Workflow in our pipeline for parameter inference of cell migration, supporting approximate Bayesian computing (ABC) and amortized neural posterior estimation (NPE). **B** Visualization of the hand-crafted summary statistics for ABC. **C** Design of neural network architectures and the corresponding training objective for the standalone summary network *s*_***ψ***_ (ABC-PM) and the joint objective for neural posterior estimator ${q}_{{{\boldsymbol{\psi }}}^{{\prime} }}$ and summary network *s*_***ψ***_ (NPE). **D** Latent-space representation of the NPE summary network obtained via LASSO-regression (*α* = 0.1).

To estimate these parameters from data, we established a simulation-based inference pipeline (Fig. 2A). This pipeline supports (i) approximate Bayesian computation sequential Monte Carlo (ABC) with hand-crafted summary statistics (Fig. 2B), (ii) ABC with neural feature extractors trained to predict posterior means (ABC-PM) (Fig. 2C), (iii) ABC with inference-tailored summaries (ABC-NPE) (Fig. 2C), and (iv) NPE using jointly trained summary networks (see “Methods”).

The four methods that are supported by our pipeline differ in how they summarize the data and approximate the posterior distribution. Sequential Monte Carlo ABC approximates the posterior iteratively by updating the proposal distribution across multiple generations until a certain stopping criterion is met, which makes it computationally demanding. In contrast, NPE uses a conditional normalizing flow combined with a summary network that is jointly trained on simulation-parameter pairs by minimizing the Kullback–Leibler divergence between the true and estimated posterior. The summary network learns a latent representation of the data that is specifically tailored for parameter inference (Fig. 2D), in contrast to hand-crafted summary statistics, which may fail to capture the full information content of the data. This motivates the use of neural networks as adaptive summary extractors. Earlier approaches trained neural networks to predict posterior means using a mean-squared error objective and then integrated these predictions into ABC as data-driven summaries^32,42^. In contrast, we reuse the summary network trained within the NPE framework directly within ABC, ensuring that the learned representations are optimized for posterior inference. Neural networks can be more sensitive to model misspecification^43,44^, whereas ABC provides the guarantee that the chosen summary statistics are optimized with respect to a defined distance metric^45^. Combining both approaches, therefore, yields a data-driven inference framework that benefits from the flexibility of neural networks while retaining the robustness guarantees of ABC.

This comprehensive pipeline allows for the assessment of the different methods for inference of Cellular Potts model parameters from DC trajectories, linking experimental migration data to the underlying stochastic processes shaped by chemokine gradients and physical barriers.

### Inference-tailored summaries enable accurate inference on simulated cell movement in complex environments

To evaluate the different methods supported by the pipeline and to assess the reproducibility of results, we performed an in silico study. Therefore, we conducted experiments using three synthetic datasets generated by the simulation model, as additional runs would substantially increase computational cost without providing further insights. For each dataset, we attempted to recover the underlying ground truth parameters, which were sampled from the prior distribution of the parameters of the CPM (see “Methods”).

**Fig. 3:**
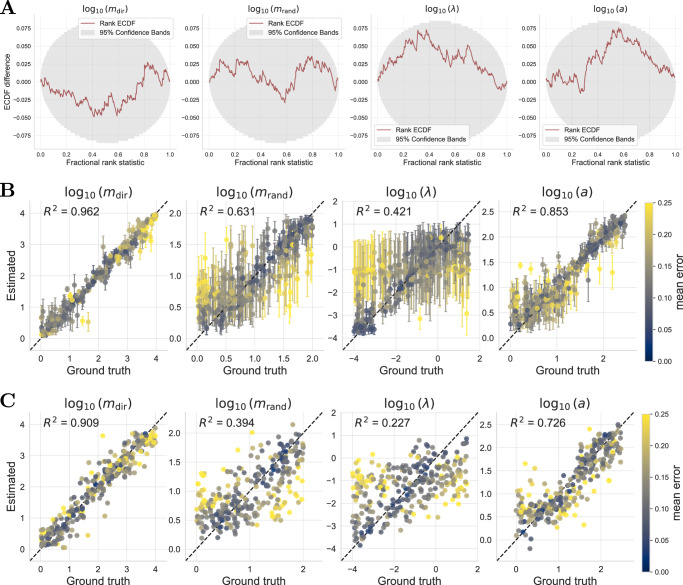
Overview of the neural network approaches and their performance on synthetic data. **A** The difference between the empirical cumulative distribution (ECDF) of the rank of the true parameter in the NPE posterior and a uniform ECDF is shown with 95% simultaneous confidence bands (red line should be in the gray area). **B** Recovery of the parameters by the neural posterior estimator (NPE) shown by the median and the median absolute deviation. **C** Recovery of the parameters by the neural network trained on the posterior mean (NPE-PM). Parameter combinations for one dataset are jointly colored with respect to the median error of the posterior.

**Fig. 4:**
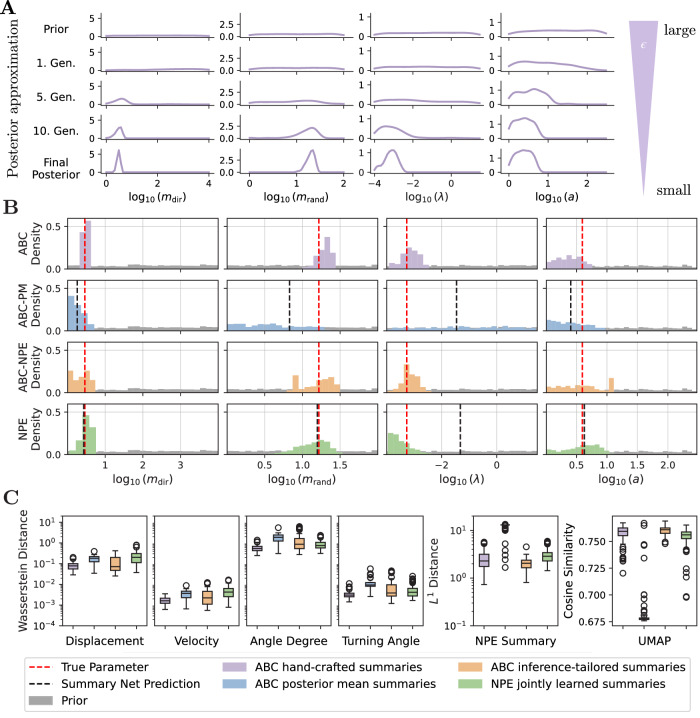
Posterior approximations and summary statistics on synthetic data. **A** Sequential update steps of ABC with hand-crafted summaries for all parameters in the cell migration model. **B** Posterior approximations using the different ABC approaches and NPE with jointly trained summaries and predictions from summary latent spaces. **C** The distance between hand-crafted summaries and the statistics of the synthetic test set was quantified using the Wasserstein distance, the *L*^1^ distance computed in NPE summary space, and cosine similarity in UMAP representation. All metrics were based on 100 simulation samples drawn from each posterior. Distributions are shown as box plots, indicating the medians, quartiles, and outliers.

The ABC methods were run until either a minimum acceptance rate of 0.01 was reached or a maximum of 15 sequential generations was completed, including a pre-calibration round, resulting in sequential updates of the posterior (Fig. 4A). In total, this required up to 432,000 simulations and runtimes between 2 and 20 h (Table 1). The assessment of the inference results revealed that all ABC approaches converged, as indicated by the low acceptance thresholds in the last generation (Supplementary Figs. 1–3). For NPE, we trained the networks on a fixed simulation budget of 32,000 simulation-parameter pairs, and since the inference procedure is amortized, the parameters can be inferred for any new dataset without retraining. To assess the convergence and calibration of the posterior in this setting, we applied simulation-based calibration (SBC) on a larger validation dataset of 300 new simulations sampled from the prior, which confirmed good calibration and showed no visible bias (Fig. 3A). SBC leverages the principle that, under a well-calibrated posterior, the rank of the true parameter within the posterior samples should follow a uniform distribution^46,47^. Such rigorous validation was not feasible for the ABC methods due to their prohibitive inference time.

**Table 1:** Comparison of the different inference methods on simulation budget and performance metrics

Method	# Simulations	Training	Inference	# Generations	NRMSE
ABC	66,000–413,000	–	2.1–19.2 h	12–16	0.23
ABC-PM	59,000–432,000	1.5 h	1.8–12.2 h	11–16	0.48
ABC-NPE	120,000–428,000	4.0 h	3.7–13.4 h	13–16	0.23
NPE	32,100	4.0 h	1 s	–	0.20

ABC-based methods were evaluated on three representative simulated datasets, whereas NPE was validated on 100 datasets. We computed the normalized root mean squared error (NRMSE). The training time accounts for both training set generation and neural network training and was measured on a high-performance computing cluster (for details, see “Methods”).

The assessment of the inference results revealed that in terms of accuracy, measured via the normalized root mean squared error (NRMSE), NPE yielded the smallest error, closely followed by ABC and ABC-NPE (Table 1). Interestingly, the neural network trained on the posterior mean achieved a smaller NRMSE of 0.18 when directly predicting the parameters on the validation data. However, when used as a feature extractor in the ABC-PM, the posterior for *m*_rand_ and *λ* remained largely uninformative, and the error was much higher, with an NRMSE of 0.48 on the three synthetic datasets. In contrast, NPE achieved better performance using far less simulations with a training duration of approximately 4 h (Table 1). Moreover, inference with NPE is essentially instantaneous because the networks are trained once and can then be readily applied to new data, enabling the efficient processing of large numbers of datasets and allowing us to analyze parameter identifiability (Fig. 3B). For instance, the identifiability assessment for the rate parameter *λ* of the waiting time revealed an overall lower posterior contraction (Supplementary Fig. 4) and a diminished parameter recovery accuracy with NPE on the validation data (Fig. 3B), while the posterior is still well calibrated (Fig. 3A). The recovery error is correlated with small values of *m*_rand_ (−0.24 Pearson correlation), and additionally the lower identifiability of *λ* may be attributed to the temporal resolution of the observations (30 s between each image), which limits the amount of informative signal available for estimating this parameter.

Our findings suggest that the ABC-PM method, which relies on the posterior mean, exhibits a systematic bias in one of the parameters: the true value of the intrinsic random motion strength *m*_rand_ frequently lies outside the high-density region of the inferred posterior distribution (Supplementary Fig. 4), despite the summary network itself showing no apparent bias during validation (Fig. 3C). In contrast, the use of hand-crafted or inference-tailored summaries led to improved parameter recovery in ABC (Table 1).

An exploratory analysis of latent summary representations revealed that one advantage of posterior mean summaries lies in their interpretability: each component corresponds directly to a model parameter, rendering the representation transparent and easily analyzable, whereas the latent summary representations learned jointly with the normalizing flow are not inherently interpretable. However, a latent-space representation of the NPE summary network obtained via LASSO-regression of true parameter values onto the simulation summary space representation reveals that half of the latent dimensions primarily focus on the influence strength of chemokine attraction *m*_dir_, with varying degrees of influence on others, indicating a degree of functional disentanglement in the learned representation (Fig. 2D). Moreover, a simple regression of the latent dimension onto the parameters already allows the prediction of the true parameters (Fig. 4B).

To better understand these findings, we examined the informativeness of the summary statistics across methods. Specifically, we computed the hand-crafted summaries, extracted the summaries from the NPE summary network, and applied a UMAP projection to simulations drawn from all four inferred posteriors. Each of these metrics provides insight into how sensitive the corresponding summaries are to variations in the simulations, while the UMAP projection, together with the cosine similarity as distance metric, offers a dimensionality reduction that is independent of the inference approach, since the metric is not part of any inference method or loss. If a cell was not observed in the simulation because it moved outside the visible window, it was not included in the corresponding statistics. When using the metric employed by ABC, the Wasserstein distance, simulations from the posteriors and synthetic test data were similar across all approaches, despite their distinct posterior distributions (Fig. 4B). However, the summaries extracted from the NPE summary network and the UMAP projection reveal notable deviations, particularly between ABC with posterior mean summaries and the other methods. Since the hand-crafted summaries exhibit smaller differences across approaches, this suggests that they are less sensitive and may be insufficiently expressive to capture the full range of informative variations of the stochastic model.

In summary, although hand-crafted and posterior mean summaries remain interpretable, they can fail to capture the variability required for reliable parameter recovery. In contrast, inference-tailored summaries adapt their representation to the data and inference task, leading to improved parameter recovery. By optimizing the summary space directly for parameter estimation, this provides a flexible and data-driven approach that enables more accurate and robust inference.

### Robust model calibration and validation of cellular dynamics on empirical data

**Fig. 5:**
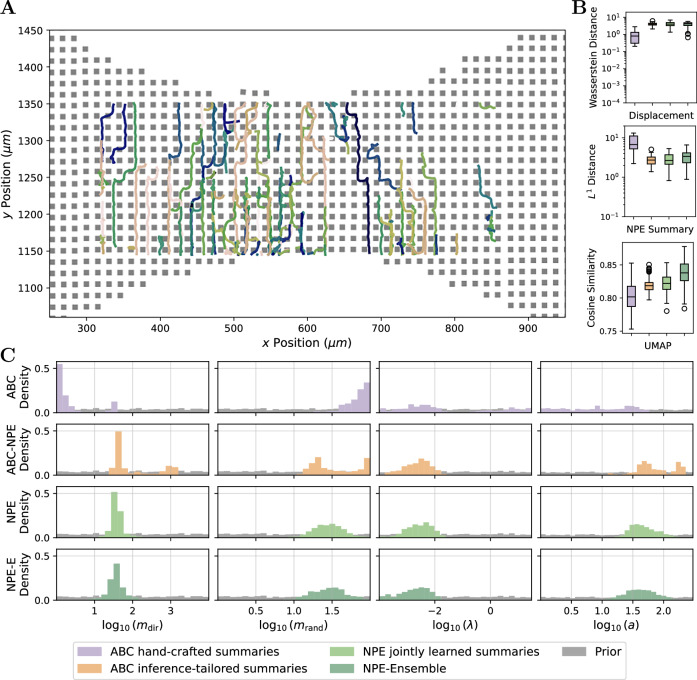
Posterior approximations and summary statistics on experimental data. **A** Experimental data (each cell in a different color) within the pillar forest are shown following the chemokine gradient upwards. **B** Distances between the hand-crafted summaries in the NPE summary space and UMAP representation were computed. All metrics are based on 100 simulation samples drawn from each posterior. Distributions are shown as box plots, indicating medians, quartiles, and outliers. **C** Posterior approximations using different ABC approaches and NPE with jointly trained summaries.

Given the validated inference pipeline, we investigated the interplay between chemokine signaling and physical constraints in DC migration using experimental data (Fig. 5A). The dataset contained 143 individual cell trajectories of varying lengths (18–120 frames per cell, with a mean duration of 33.2 min), reflecting heterogeneity in tracking durations across the experiment.

ABC was performed using both hand-crafted and inference-tailored summary statistics. Both approaches converged after 13 generations, as indicated by the small final acceptance thresholds, requiring approximately 200,000–300,000 simulations and 27–50 h of runtime (Supplementary Fig. 5). In addition, NPE was employed, including an ensemble of three independently trained networks (NPE-E), to obtain a more robust estimate that is less sensitive to variations in individual network training.

To assess the differences between the approaches, we compared the hand-crafted summary statistics on posterior simulations, the *L*^1^ distance in the NPE summary space, and the cosine similarity in the UMAP projection. Among the hand-crafted summaries, which are directly minimized by ABC, only the displacement feature shows a noticeably smaller distance for ABC with hand-crafted summaries (Fig. 5B); the remaining summaries yield highly similar distances across all methods (Supplementary Fig. 5C). In contrast, the *L*^1^ distance, which is directly minimized only by ABC-NPE, and cosine similarity metrics indicate that the predictions from the NPE-based methods are generally closer to the observed data. The ensemble NPE achieved the highest cosine similarity, whereas both the single and ensemble NPEs exhibited nearly identical *L*^1^ distances. Because the UMAP projection was not used during training and inference in any method, this metric offers an independent measure of posterior predictive quality. As an additional robustness check, we exploited the amortization property of the NPE model to infer parameters from truncated datasets, since our summary network accommodates a variable number of cells. We observed that when using data from approximately 50 cells, the posterior estimates become stable and consistent with those obtained from the full dataset (Supplementary Fig. 7A). Furthermore, we controlled for potential model misspecification (Supplementary Fig. 7B).

The posterior distribution obtained from the ensemble was slightly more conservative than the one obtained from the single NPE model, but still indicating consistency across the training runs (Fig. 5C). In contrast, the inference-tailored ABC posterior samples show slightly greater uncertainty and hint at a potentially multimodal posterior, although its median aligns with that of the NPE-based estimates (Fig. 5C). Notably, ABC using hand-crafted summaries yielded a markedly different posterior, with a distinct shift in the median and signs of bimodality (Fig. 5C). For instance, the larger mode in the inferred chemokine attraction strength *m*_dir_ aligns with the dominant mode found in the NPE posteriors and the smaller mode seems to be the key driver in increasing the uncertainty of the other parameters (Supplementary Fig. 6).

The median estimated target cell area from NPE-E is *a* = 41.73 μm^2^ (Fig. 5C), corresponding to an effective circular diameter of 7.29 μm. This smaller apparent size indicates that the cells respond to confinement within the pillar forest, as their estimated diameter is below both the pillar spacing of 10 μm and the expected size of mature DCs (10 − 15 μm) [ref. ^48^, BNID 113239]. The reduced diameter also reflects the limitations of a circular approximation, as BMDCs are highly deformable and frequently adopt elongated or irregular shapes under confinement. In the Cellular Potts model, the target area *a* acts as a soft constraint on compactness, which does not capture vertical deformations but allows realistic in-plane deformation and interaction with the pillars. Additionally, the median estimated rate parameter is *λ* = 2.1 ms^−1^, corresponding to a mean waiting time of 7.9 min, which is more than one order of magnitude larger than the time of 30 s between two images.

In conclusion, our results demonstrate the robustness and flexibility of our Cellular Potts model in capturing key cellular dynamics. By integrating posterior inference with neural summaries, we successfully identified critical parameters that govern cell migration.

### Simulation study shows the modulating role of physical obstacles and persistence in cell migration

To assess how physical structures modulate migratory behavior, we used our computational model and inference pipeline to investigate the impact of obstacles on cell movement. Specifically, we aimed to disentangle the independent contributions of chemokine guidance and persistence in random motility.

**Fig. 6:**
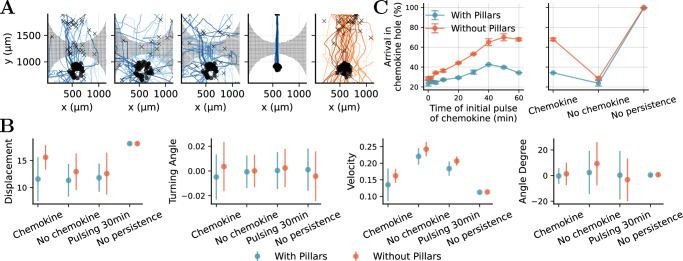
Simulation study shows the modulating role of obstacles. The trajectory of 50 random cells (left) and the distribution of the turning angles (of all cells) (right) are shown. The endpoints are marked with “x” if visible in the selected area. **A** Simulations under different conditions. From left to right: setup mirroring the experiment with structural obstacles and a constant chemokine gradient; setup without a chemokine; setup in which the chemokine is active only for 30 min; setup without persistence in the random walk but with a constant chemokine; setup without pillars but with persistence in the random walk and a constant chemokine. **B** Median and absolute deviation of the summary statistics for the different conditions. **C** Median and absolute deviation of the percentage of cells reaching the chemokine hole after an initial pulse and under different conditions with and without pillars.

Based on the posterior median of the model parameters from the NPE ensemble, we simulated cell migration under four distinct conditions: with and without a chemokine gradient that was constant over time, with an initial chemokine signal lasting only 30 min, and with suppressed persistence in random movement (intrinsic random movement was set to *m*_rand_ = 0) but a constant chemokine gradient (Fig. 6A). Simulations were sampled at a higher resolution of 10-s intervals and also recorded outside the visible window of the experimental setting, enabling controlled comparisons of cell migration with and without the pillar forest.

The results demonstrate that persistence is a key driver of cell dispersion (Fig. 6). Even in the absence of directional guidance through the chemokine gradient, cells with persistent movement spread widely throughout the environment, but do not necessarily reach the chemokine hole (Fig. 6A). In contrast, when persistence is removed, migration is spatially constrained and directionally limited as indicated by the low variance of the angle degree (Fig. 6B).

The pillar forest further modulates this behavior by imposing spatial constraints that can trap cells.

This phenomenon became even more apparent when analyzing the number of cells that reached the upper clearing of our experimental setup. Simulations where the chemokine signal is active only at the first 50–75% of the simulation time show a higher proportion of successful arrivals compared to scenarios with constant guidance (Fig. 6C). The simulations show that the presence of pillars hinders migration, as fewer cells reach the source, and that this difference increases with the duration of the chemokine pulse (Fig. 6C). While without the pillar forst the velocity of the cells increase compared to when the pillars are present, the displacement increases less if the chemokine is not or only partly present compared to the situation with a constant chemokine presence (Fig. 6B). Thus, prolonged chemokine exposure can keep cells trapped within the pillar forest. In contrast, when directional persistence is removed, cells are no longer confined, even in the presence of obstacles. This indicates that it is the combination of chemokine signaling and persistence that can lead to cell entrapment. Our experimental setup does not allow us to directly observe and thus verify this outcome, as our imaging is limited to the pillar forest region and does not extend to the final source location. Nevertheless, a similar effect may occur in vivo, potentially because of signal decay or attenuation near the source. This counterintuitive result highlights that temporally modulated guidance cues can facilitate more efficient exploration in the presence of obstacles.

In summary, the simulations show that the Cellular Potts model captures how physical obstacles reshape migration dynamics in our setup, not only by slowing cells, but also by altering the directionality and dispersion of their paths. While chemokines impose a directional bias, obstacles introduce nonlinear constraints that modify or obscure this signal in the observable data.

## Discussion

A quantitative understanding of cell migration is essential across diverse domains, including immunology, oncology, and tissue engineering, where directed cell movement plays a central role in physiological and pathological processes. Despite its importance, methods for inferring the parameters of mechanistic models of migration from experimental data remain limited, especially in spatially structured or noisy microenvironments, where hand-crafted summaries may fail to capture the relevant dynamics.

The findings of this study provide a foundation for researchers to enhance modeling approaches for biological systems. Specifically, our model is able to capture the substantial impact of spatial obstacles on cell migration, as shown experimentally in previous work^40^. Additionally, we demonstrate that the learned summaries exhibit superior performance compared to traditional summary statistics in the context of simulation-based inference. Our simulation study further revealed that persistent cell motion plays a critical role in overcoming spatial constraints and that temporally modulated chemokine cues can facilitate dispersal in obstacle-rich environments.

The pillar forest used in our simulations provides a well-controlled and interpretable setting for uncovering the fundamental principles of cell migration. Building on our framework, future studies could further validate our model and examine how cells behave in spatially and temporally complex environments, where guidance cues may be transient, competing, or context-dependent. Such scenarios would require extending the current model, for example, by coupling the Cellular Potts model with a partial differential equation to capture environmental dynamics, which would introduce additional parameters to be inferred. It remains an open question how cells resolve directional conflicts in the presence of multiple chemotactic sources, or how robust their migratory behavior is in the face of spatial noise or fragmented gradients. Addressing these challenges would advance our understanding of how immune cells navigate physiologically realistic environments and flexibly respond to complex environmental stimuli.

The utilization of learned summaries in ABC shares similarities with other approaches that employ an estimator trained on pairs of simulations and parameters to predict the posterior mean or learn a summary based on an autoencoder^35,49^. Yet, the latent space of these autoencoders is not specifically tailored for inference but for reconstruction of the data. The posterior mean estimators are then used in the context of ABC to generate summary statistics^32,42,50–52^. However, we demonstrated that the posterior mean was not sufficiently informative. Our results suggest that ABC-PM exhibits a systematic bias, despite the summary network itself showing no apparent bias during validation. We observe this behavior only on parameters with possible non-identifiabilities, which predominantly occur when the random motility parameter *m*_rand_ is small, indicating that motion is almost entirely driven by chemotactic attraction. In these cases, the waiting-time distribution becomes less informative because cells no longer follow a persistent random walk, leading to an overestimation of *λ*. This discrepancy likely arises because predicting only the posterior mean does not adequately capture the uncertainty inherent in the data, resulting in biased estimates. Therefore, it was recently proposed to append noise estimates or higher-order moments of the parameters to the posterior mean^34,35^. However, this approach remains a type of manual feature engineering. Moreover, although the neural network architecture is identical for both the posterior mean and inference-tailored summaries, joint training with the normalizing flow removes the constraint that the output dimensionality of the summary network matches the parameter space, thereby enabling greater representational flexibility.

Adaptive distances based on hand-crafted summaries have been extensively studied^33,34^. Yet, identifying the optimal distance measure depends heavily on the informativeness of the chosen statistics, which may fail to capture the necessary information. In contrast, the joint learning of summaries with a neural posterior estimator ensures that the summaries are maximally informative and eliminates the need for additional upfront simulations. Moreover, our findings indicate that ABC requires 10 times more simulations to attain comparable accuracy, thereby highlighting the efficacy of NPE, which is consistent with previous findings^38,53^. Normalizing flow architectures for NPE typically perform well for up to several dozen parameters, and recent developments in flow matching enable efficient inference in substantially higher-dimensional settings^54^. As cells are heterogeneous in size and even have specific shapes depending on the chemical gradient^55^, a further direction could be to quantify individual differences between cells using an amortized hierarchical framework^56,57^.

Instead of applying the Wasserstein distance to the hand-crafted summaries, the distance has been utilized directly on the observed data^58^. However, this approach has not been generalized to a spatial context with a varying number of observations and therefore cannot be applied in our context. In general, if the quantiles of the statistic (or the data) do not vary significantly as the parameter changes, the Wasserstein distance will not change in a meaningful manner and thus cannot serve as an accurate posterior approximation^59^.

An opportunity to extend our model lies in its current assumption of a homogeneous and static environment. While this simplification has provided clear insights into core migration behaviors, it does not capture the dynamic, feedback-rich nature of many biological systems, where cells actively remodel their surroundings and chemokine gradients do not follow a Gaussian distribution. For example, the chemokine concentration diffuses over time, migrating cells may reshape local chemotactic gradients^60,61^, restructure the extracellular matrix^62^, or exert mechanical forces, establishing feedback loops that influence both their own motility and collective behavior^63^. Moreover, DC migration is tightly integrated with their immunological functions, such as antigen capture, cytokine production, and T-cell priming, and is influenced by a complex interplay of extracellular cues and intracellular signaling pathways^64^. Incorporating such bidirectional cell-environment interactions into future models offers the potential for more faithful representations of the adaptive and co-evolving dynamics that characterize living tissues. Experimental evidence further shows that migrating cells maintain their speed under strong confinement but lose directional control depending on pillar spacing^65^, and that dendritic cells require stable spatial chemokine gradients to maintain directional persistence, as transient or oscillatory cues rapidly disrupt cytoskeletal polarization and migration^66^. This highlights that dynamic environmental signals may not only fail to support chemotaxis but can actively destabilize it, reinforcing the need to jointly model spatial and temporal features.

The findings reveal that learned summaries, tailored specifically for inference, exhibit superior performance compared to hand-crafted summary statistics and provide more information than summaries based on the posterior mean. This enhancement in performance can be attributed to the adaptability of neural networks, which automatically extract relevant features from data, thereby eliminating the need for manually designed summaries. Instead, these networks learn features that are specifically relevant to the inference task. By leveraging this approach, researchers can develop more complex models and calibrate them effectively with experimental data, paving the way for deeper insights into the fundamental mechanisms of cell migration.

## Methods

In this study, we analyzed the movement of bone marrow-derived DCs (DCs) in complex microenvironments, where both chemical signaling and physical barriers influence their movement.

### Experimental procedures

DCs are confined in an environment with 7 ng of the chemokine (C-C motif) ligand 19 (CCL19), which binds to the C-C chemokine receptor type 7 (CCR7) expressed on the surface of mature DCs^67^. To create physical obstacles, the area between the two confining surfaces was intersected by pillars with defined gaps: a “pillar forest”^40^ (see Fig. 1). The presence of these obstacles introduces physical complexity, compelling cells to traverse the gaps of 10 μm. This adds a layer of mechanical challenge to their movement, as the cells constantly encounter pillars on their migratory path.

#### Dendritic cell culture from mouse bone marrow

Femurs and tibias from the legs of 6–12 week-old C57BL/6J mice were removed and placed in 70% ethanol for 2 min. Bone marrow was flushed with PBS using a 26-gauge needle. 2 × 10^6^ cells were seeded per 100 mm Petri dish containing 9 mL of complete medium (Roswell Park Memorial Institute 1640 supplemented with 10% Fetal Calf Serum (FCS), 2 mM L-Glutamine, 100 U/mL Penicillin, 100 μg mL^−1^ Streptomycin, and 50 μM *β*-mercaptoethanol; ThermoFischer Scientific) and 1 mL Granulocyte-Monocyte Colony Stimulating Factor (GM-CSF, supernatant from hybridoma culture). On days 3 and 6, complete medium supplemented with 20% GM-CSF was added to each dish. To induce maturation, the cells were stimulated overnight with 200 ng mL^−1^ lipopolysaccharide (LPS) and used for experiments on days 8 and 9. Alternatively, DCs were frozen in FCS containing 10% DMSO on day 7. For experimental use, the cells were thawed the day before the experiment and stimulated with 200 ng mL^−1^ LPS overnight.

#### Photomask patterning

Photomasks were drawn in CorelDRAW and converted to Gerber in LinkCad. Photomasks were fabricated at JD Photodata (UK). These photomasks were used to define microscale patterns on a photoresist-coated silicon wafer during UV lithography. The transparent and opaque regions of the mask control the areas where UV light reaches the photoresist, enabling precise pattern transfer. To produce the master wafer, a 4-inch wafer was first baked at 110 °C for 5 min. 6005 TF SU8 was spun up to 3000 RPM with an acceleration of 300 RPM/s for 30 s. The wafer was then baked at 110 °C for 5 min. The wafer was exposed to 100 mJ/cm^2^ UV light through the photomask, then baked for 5 min at 110 °C, and then developed in SU8 developer. As a final step, the wafer was baked at 135 °C for 5 min to permanently harden the SU8. The height of the wafer was approximately 5 μm.

#### Pillar forest migration assay

Microfabricated chips were produced by pouring a polydimethylsiloxane (PDMS) mixture (silicon rubber and curing agent at a ratio of 10:1 by weight) onto a silicon wafer with a previously designed photomask pattern. This was incubated at 80 °C overnight after removing air bubbles by placing the PDMS in a vacuum desiccator. The PDMS was separated from the wafer, and single chips were cut out using a surgical blade. Next, two holes (diameter of 1.5 mm) on each side of the pillar forest area were punched at a distance of 1 mm, and the PDMS was bound upside-down to a glass-bottom dish. To activate the surface, both the PDMS chip and dish were cleaned with plasma before assembly. To equilibrate the chip, it was flushed with complete medium and incubated at 37 °C, 5% CO_2_ for at least 1 h. Afterward, the medium was removed from the two holes, and they were filled with 7 μL CCL19 solution at a concentration of 1 g mL^−1^ on one side and 3 × 10^4^ cells in complete medium on the opposite side. Additionally, dextran was added to the CCL19 solution (1:1000) to visualize the chemokine gradient. BMDCs were stained with NucBlue (Invitrogen) and concentrated to 6.25 × 10^6^ cells/mL in advance.

#### Microscopy and image analysis

After a 2–2.5 h incubation at 37 °C, 5% CO_2_, samples were imaged with an inverted wide-field Nikon Eclipse Ti-2E microscope in a humidified and heated chamber at 37 °C and 5% CO_2_ (Ibidi Gas Mixer), equipped with a Plan-Apochromat 20×/0.8 air objective, a DS-Qi2 camera, and a Lumencor Spectra X light source (390 nm, 475 nm, 542/575 nm; Lumencor). Movies were taken for 2 h, recording one image every 30 s. Videos were analyzed using Fiji^68^. Cell nuclei tracking was performed manually or automatically using the Fiji plugin TrackMate^69^. However, owing to the microscope setup, tracking was only possible within the pillar forest between the two clearings.

### Model description

To simulate the squeezing of cells past obstacles, a model with a representation of variable cell shape is required. In this paper, an extended Cellular Potts model (CPM) based on the classical formulation by Graner and Glazier^11^ was used. It provides an explicit representation of cell shape and, through extensions, can depict both undirected cell movement and the response to chemoattractants (see Fig. 1C). The classical CPM represents cells as connected domains of lattice sites on a discrete three-dimensional grid and evolves stochastically according to a Metropolis update rule. At each iteration, a lattice site *i* occupied by cell *σ*(*i*) and one of its neighbors *j* belonging to cell *σ*(*j*) are chosen at random, and a copy attempt *σ*(*j*) → *σ*(*i*) is proposed. The move is accepted with probability $\min \{1,\exp (-\Delta H/T)\}$, where Δ*H* is the change in effective energy assigned to the system, and *T* controls the amplitude of stochastic fluctuations.

The classical CPM energy function is given by

1 $$H=\mathop{\sum }\limits_{\langle i,j\rangle }{J}_{\sigma (i),\sigma (j)}(1-{\delta }_{\sigma (i),\sigma (j)})+{\lambda }_{v}\mathop{\sum }\limits_{c=1}^{N}{({v}_{c}-a)}^{2}.$$

where *σ*(*i*) denotes the cell at site *i*. The first (adhesion) sum runs over neighboring lattice sites and penalizes interfaces between distinct cells or between a cell and the medium, where *J* is the coefficient determining the adhesion. The second (volume) sum runs over all *N* cells and enforces approximate conservation of the target volume *a* for each cell, thereby capturing differential adhesion and surface tension, where *λ*_*v*_ determines the strength of the volume constraint. A proposed update then changes the energy function by the difference in the energy of these two terms Δ*H* = Δ*H*_adhesion_ + Δ*H*_volume_. The combined effects of surface tension and volume conservation allow the model to capture mechanical interactions with neighboring cells and the extracellular matrix. In dense environments, deviations from the preferred volume can generate mechanical pressure, resulting in tissue-level reorganization.

To model chemotactically guided cell migration, we extended the basic CPM with two additional components: directional movement in response to chemokine gradients and intrinsic persistent random motion. To bias cell movement toward higher concentrations, the chemotactic signal is integrated into the energy function via an additive term

2 $$\Delta {H}_{{\mathrm{chemotaxis}}}={m}_{{\mathrm{dir}}}\cdot ({f}_{{x}_{i}}-{f}_{{x}_{j}}),$$

where ${f}_{{x}_{i}}$ and ${f}_{{x}_{j}}$ are the local chemoattractant concentrations at the occupied and empty lattice sites, respectively, and *m*_dir_ controls the strength of chemotactic attraction^70^. The update is energetically favored if the move leads to a position with a higher concentration.

To account for undirected and exploratory cell movements, we implemented a persistent random walk. This implementation resembles a run-and-tumble process and reflects experimentally observed persistent random motion^16^. Each cell was assigned a preferred migration direction that was periodically updated. The energy contribution for a proposed move with induced displacement is given by another additive term to the energy function *H*:

3 $$\Delta {H}_{{\mathrm{persistence}}}=-{m}_{{\mathrm{rand}}}\cdot {v}_{c}\cdot \langle {(\cos \alpha ,\sin \alpha )}^{T},{{\boldsymbol{s}}}_{c}\rangle ,$$

where *α* is the current migration angle, *v*_*c*_ is the current area of the cell *c*, *m*_rand_ is the strength of intrinsic motility, and 〈⋅, ⋅〉 denotes the inner product. The scalar product penalizes deviations from the preferred direction and thus reproduces the observed persistence of cell motion. Experimental and theoretical studies show that cell migration often follows stochastic dynamics with exponentially distributed waiting times between directional changes^71^. Accordingly, for each cell, the angle *α* is updated after a waiting time ${t}_{\alpha } \sim Exp(\lambda )$ with rate *λ*, and a new direction is sampled uniformly from ${\mathcal{U}}[0,2\pi ]$.

Since only the energy difference Δ*H* determines the system’s evolution, the total energy change associated with a proposed move combines all classical and extended contributions:

4 $$\Delta H=\Delta {H}_{{\mathrm{adhesion}}}+\Delta {H}_{{\mathrm{volume}}}+\Delta {H}_{{\mathrm{chemotaxis}}}+\Delta {H}_{{\mathrm{persistence}}}.$$

Overall, the modified CPM integrates classical mechanical interactions with chemotactic and stochastic motility components, enabling the simulation of both directed and random cell migration in complex multicellular environments.

Because the cells are comparatively flat in the considered experimental setup, a 2D model was used to capture the exploration details without adding unnecessary complexity. We used an image to position a dense array of pillars between the two ends of the migration area, thereby reproducing the spatial constraints of our experimental setup. Proposed update steps onto the pillars are rejected, preventing cells from entering the spatial obstacles. The model was built using MorpheusML, an XML format for encoding multiscale models and multicellular biological systems^16^. Spatial resolution is given by the resolution of our image of the pillar forest and is therefore set to 1.31 μm per node length. Due to the complex parameter scaling in the Cellular Potts model, the inferred cell migration parameters are optimized only for this resolution and cell size, yielding reliable, biologically relevant results. To emulate the experimental setting, we simulated cell movement in the entire area and extracted the coordinates of cells in the pillar forest at 30-s intervals. The chemokine gradient was modeled as a time-independent Gaussian distribution with a peak concentration of 7 × 10^6^ μm^−2^ centered at the chemokine hole and a standard deviation of 550 μm. This choice reflects typical diffusion distances and ensures a smooth and biologically meaningful gradient.

In summary, the parameters to be inferred from the data in this study are

5 $${\boldsymbol{\phi }}={[{\log }_{10}({m}_{{\mathrm{dir}}}),{\log }_{10}({m}_{{\mathrm{rand}}}),{\log }_{10}(\lambda ),{\log }_{10}(a)]}^{T}$$

with their corresponding priors provided in Table 2. Weakly informative uniform priors were specified on the logarithmic scale to ensure that the inference is primarily data-driven, and bounds were selected based on biological constraints or the computational feasibility of the simulations. The lower bound of the movement parameters was chosen as the minimal strength that ensured cells appeared within the visible window of the experimental setup, while smaller values resulted in static or invalid simulations. For the cell area parameter, a broad prior was used to reflect biological variability in the size and spreading behavior of bone marrow-derived DCs across different microenvironments. Additional technical constants used in the CPM, such as the coefficient of adhesion *J*, the volume constraint *λ*_*v*_, and the fluctuation amplitude *T*, are documented in the model file, provided here: https://identifiers.org/morpheus/M6342.

**Table 2:** Parameters to estimate in the cell migration model

Parameter	Description	Prior
${\log }_{10}({m}_{dir})$	Influence of chemokine attraction	${\mathcal{U}}[0,4]$
${\log }_{10}({m}_{rand})$	Strength of intrinsic random movement	${\mathcal{U}}[0,2]$
${\log }_{10}(\lambda )$	Rate of the waiting-time distribution (s^−1^)	${\mathcal{U}}[-4,1.5]$
${\log }_{10}(a)$	Area of cells (μm^2^)	${\mathcal{U}}[0,2.5]$

Parameters were transformed to a logarithmic scale and had uniform priors.

### Hand-crafted summary statistics

The dynamics of the model depend on several unknown parameters that must be estimated from the available data. We employ ABC using hand-crafted summary statistics that were built specifically for this model, referred to as *ABC with hand-crafted summary statistics*.

Each simulation contains multiple cell tracks of varying lengths, as cells could move out of the observation area or leave the 2D plane. Because the trajectory of a cell is governed by stochastic movement, direct matching of a simulated cell to a real cell is not possible. Instead, summary statistics that characterize individual cell movement were constructed. These statistics were computed for each cell, and the distributions of these statistics were compared between the simulation and observed data. This approach eliminates the need for direct track matching and allows for flexibility in the number of simulated cells. However, for reliable parameter inference, the number of cells should not deviate too much, as crowded and sparse environments differ significantly; for example, cells may block each other more frequently in a crowded environment.

We constructed four different widely used summary statistics to describe different aspects of cell movement^72,73^. Denoting the position of a cell *c* ∈ {1, …, *N*} as ${{\boldsymbol{X}}}_{i}^{c}=({x}_{i}^{c},{y}_{i}^{c})$ at time step *i* for *i* ∈ {1, …, *T*^*c*^}, we define the following statistical quantities. Note that the time steps of each cell are relative to the first time they were observed.

- *Displacement (D)* measures the normalized squared distance traveled by the cell *c* over the observed time:
  6 $${{\mathrm{D}}}^{c}=\frac{1}{\sqrt{{T}^{c}}}\sqrt{{({x}_{{T}^{c}}^{c}-{x}_{1}^{c})}^{2}+{({y}_{{T}^{c}}^{c}-{y}_{1}^{c})}^{2}},$$
  where *T*^*c*^ is the number of observed time steps for each cell. We scaled the displacement by time so that different observed sequence lengths could be handled. For a standard random walk, the expected translation distance after *T* steps is of the order of $\sqrt{T}$.
- *Velocity (V)* is the average speed of a cell *c* and is expressed as the mean over all time steps
  7 $${{\mathrm{V}}}^{c}=\frac{1}{{T}^{c}-2}\mathop{\sum }\limits_{i=2}^{{T}^{c}}\frac{1}{\Delta t}\sqrt{{({x}_{i}^{c}-{x}_{i-1}^{c})}^{2}+{({y}_{i}^{c}-{y}_{i-1}^{c})}^{2}},$$
  where Δ*t* = 30 s is the interval at which the cells were observed.
- *Turning angle (TA)* quantifies the mean change in directionality of a cell *c* between consecutive movements:
  8 $${{\mathrm{TA}}}^{c}=\frac{1}{{T}^{c}-3}\mathop{\sum }\limits_{i=3}^{{T}^{c}}({\theta }_{i}^{c}-{\theta }_{i-1}^{c}),$$
  where $${\theta }_{i}^{c}=\arctan 2({x}_{i}^{c}-{x}_{i-1}^{c},{y}_{i}^{c}-{y}_{i-1}^{c})$$.
- *Angle degree (AD)* captures the average absolute direction between two consecutive positions:
  9 $${{\mathrm{AD}}}^{c}=\frac{1}{{T}^{c}-2}\mathop{\sum }\limits_{i=2}^{{T}^{c}}{\theta }_{i}^{c}.$$

To compute the acceptance criterion in ABC, we used the Wasserstein-1 distance to calculate the distance between the samples of the distributions of the summaries of the simulations and the summaries of the experimental data. The Wasserstein distance quantifies the cost of transforming one distribution into another, thereby providing a comprehensive measure of discrepancy (see ref. ^74^ for a detailed introduction), and remains well-defined even when the number of observations differs across simulations. By comparing the distributions of the summary statistics for the simulated and observed cells, we captured the differences across the entire dataset, avoiding the matching of trajectories. To integrate multiple statistics while minimizing the overreliance on any single summary, the Wasserstein distance was computed separately for each statistic. To normalize the contribution of each summary, the distance is weighted by the inverse of the difference between the largest and smallest statistics, as suggested in ref. ^75^. These weights were calculated from simulations of a pre-calibration population with parameters sampled from the prior. Finally, the weighted distances are aggregated by summing them up.

### Neural posterior estimation with learned summary statistics

NPE methods leverage neural networks to learn mappings from data to model parameters and can integrate learned summary networks that automatically extract relevant features from the data^37^. Instead of relying on hand-crafted summary statistics, we used a summary network *s*_***ψ***_ with trainable parameters ***ψ***, designed to handle variable-length inputs, both in terms of the number of observed time points per cell and the number of cells per experiment. To extract informative features from each individual cell trajectory, we applied a combination of one-dimensional convolutional layers and a recurrent neural network, which is a standard architecture for time-series processing. To aggregate across multiple cells, we employed an attention-based pooling mechanism that produced a fixed-length summary vector while preserving flexibility with respect to the cell count (see Fig. 2C and Implementation for details). The summary network is then utilized to learn a *d*-dimensional representation ${s}_{{\boldsymbol{\psi }}}({\boldsymbol{X}})\in {{\mathbb{R}}}^{d}$ of the observed data points ${\boldsymbol{X}}=\{{{\boldsymbol{X}}}^{c}(t)\in {{\mathbb{R}}}^{{T}^{c}\times 2}| c\in \{1,\ldots ,N\}\}$.

The summary statistics serve as inputs to a conditional normalizing flow, which transforms the posterior into a simpler density from which we know how to sample. This method allows efficient and accurate sampling and density evaluation^76,77^. Let ***z*** be a latent variable described by a multivariate normal distribution *p*(***z***). The model parameters ***ϕ*** are mapped to these latent variables conditional on the data *s*_***ψ***_(***X***) by a normalizing flow ${f}_{{{\boldsymbol{\psi }}}^{{\prime} }}({\boldsymbol{\phi }},{s}_{{\boldsymbol{\psi }}}({\boldsymbol{X}}))={\boldsymbol{z}}$. This invertible transformation is parameterized by ${{\boldsymbol{\psi }}}^{{\prime} }$ and, by construction, has a tractable Jacobian. The approximation ${q}_{{{\boldsymbol{\psi }}}^{{\prime} }}$ to the target density *p*(***ϕ***∣*s*_***ψ***_(***X***)) is given by the change-of-variables formula

10 $${q}_{{{\boldsymbol{\psi }}}^{{\prime} }}({\boldsymbol{\phi }}| {s}_{{\boldsymbol{\psi }}}({\boldsymbol{X}}))=p({\boldsymbol{z}}={f}_{{{\boldsymbol{\psi }}}^{{\prime} }}({\boldsymbol{\phi }},{s}_{{\boldsymbol{\psi }}}({\boldsymbol{X}})))| \det {J}_{{f}_{{{\boldsymbol{\psi }}}^{{\prime} }}}({\boldsymbol{\phi }},{s}_{{\boldsymbol{\psi }}}({\boldsymbol{X}}))| .$$

If we know ${f}_{{{\boldsymbol{\psi }}}^{{\prime} }}$, we can sample from the posterior by sampling ***z*** ~ *p*(***z***) and applying ${f}_{{{\boldsymbol{\psi }}}^{{\prime} }}^{-1}({\boldsymbol{z}},{s}_{{\boldsymbol{\psi }}}({\boldsymbol{X}}))={\boldsymbol{\phi }}$.

To train the summary network *s*_***ψ***_ jointly with the conditional normalizing flow ${f}_{{{\boldsymbol{\psi }}}^{{\prime} }}$, we follow the work of^78^ and minimize the Kullback–Leibler divergence between the true and approximate posterior distributions:

11 $$\begin{array}{rcl}{({\boldsymbol{\psi }},{{\boldsymbol{\psi }}}^{{\prime} })}^{* } & = & \mathop{{\mathrm{arg}}\,{\mathrm{min}}}\limits_{({\boldsymbol{\psi }},{{\boldsymbol{\psi }}}^{{\prime} })}\,{{\mathbb{E}}}_{p({\boldsymbol{X}})}[{\mathrm{KL}}(p({\boldsymbol{\phi }}| {s}_{{\boldsymbol{\psi }}}({\boldsymbol{X}}))| | {q}_{{{\boldsymbol{\psi }}}^{{\prime} }}({\boldsymbol{\phi }}| {s}_{{\boldsymbol{\psi }}}({\boldsymbol{X}})))]\\ & = & \mathop{{\mathrm{arg}}\,{\mathrm{min}}}\limits_{({\boldsymbol{\psi }},{{\boldsymbol{\psi }}}^{{\prime} })}\,\iint -p({\boldsymbol{X}},{\boldsymbol{\phi }})\log {q}_{{{\boldsymbol{\psi }}}^{{\prime} }}({\boldsymbol{\phi }}| {s}_{{\boldsymbol{\psi }}}({\boldsymbol{X}}))\,{\rm{d}}{\boldsymbol{X}}\,{\rm{d}}{\boldsymbol{\phi }}\\ & \approx & \mathop{{\mathrm{arg}}\,{\mathrm{min}}}\limits_{({\boldsymbol{\psi }},{{\boldsymbol{\psi }}}^{{\prime} })}\,\frac{1}{S}\mathop{\sum }\limits_{s=1}^{S}-\log {q}_{{{\boldsymbol{\psi }}}^{{\prime} }}({{\boldsymbol{\phi }}}^{(s)}| {s}_{{\boldsymbol{\psi }}}({{\boldsymbol{X}}}^{(s)})).\end{array}$$

The integral is approximated using *S* samples from the joint distribution *p*(***X***, ***ϕ***). These samples are obtained by sampling from the prior distribution ***ϕ*** ~ *p*(***ϕ***) and simulating the model ${\mathcal{M}}({\boldsymbol{\phi }})$. Using the transformation (10), the approximation in (11) can be efficiently evaluated. After training, we performed SBC checks to validate the convergence of the neural posterior estimator (Fig. 3A).

### Approximate Bayesian computing with inference-tailored summary statistics

Studies have shown that NPE is sensitive to model misspecification and can potentially provide biased estimates^43,44^. In contrast, ABC is sensitive to the choice of the summary statistics but converges to the optimal parameter under this summary^45^. To address these limitations, we consider a combined approach in this study: hand-crafted summaries of ABC are replaced by trained summary networks, as in NPE. The learned summaries are computed for each simulation by passing the data through the trained summary network. The *L*^1^ distance is utilized in place of the Wasserstein distance on the learned summaries, as the objective is no longer to compare distributions but rather to enhance robustness against outliers^79^. This approach is referred to as *ABC with inference-tailored summaries* or ABC-NPE.

### Approximate Bayesian computing with posterior mean summary statistics

Previous work suggested training a neural network ${\widetilde{s}}_{{\boldsymbol{\psi }}}({\boldsymbol{X}})\in {{\mathbb{R}}}^{\dim ({\boldsymbol{\phi }})}$ on data ***X*** to approximate the posterior mean ${\mathbb{E}}[{\boldsymbol{\phi }}| {\boldsymbol{X}}]$ of the model parameters ***ϕ*** and then using the trained network to compute summary statistics of the data^32,42^. In this approach, the summary network is trained by optimizing the mean squared error objective

12 $${{\boldsymbol{\psi }}}^{* }=\mathop{{\mathrm{arg}}\,{\mathrm{min}}}\limits_{{\boldsymbol{\psi }}}\frac{1}{S}\mathop{\sum }\limits_{s=1}^{S}| | {\widetilde{s}}_{{\boldsymbol{\psi }}}({{\boldsymbol{X}}}^{(s)})-{{\boldsymbol{\phi }}}^{(s)}| {| }_{2}^{2}.$$

Hence, the output dimension of the network is fixed to the number of parameters. The network is also trained on pairs of parameters and simulations, and can therefore be applied to unseen data to estimate the posterior mean directly. When the acceptance threshold *ϵ* is sufficiently small, this approach, referred to as *ABC with posterior mean summaries* (ABC-PM), converges to a posterior with the same mean as the exact posterior^32,34^. As before, we use the *L*^1^ distance here.

### Error metrics for posterior and simulated data

We computed the NRMSE for each parameter across *N* datasets and aggregated the error over posterior samples using the median as follows:

$${\mathrm{NRMSE}}=\frac{1}{\max ({{\boldsymbol{\phi }}}_{{\mathrm{true}}})-\min ({{\boldsymbol{\phi }}}_{{\mathrm{true}}})}{\mathrm{median}}(\sqrt{\frac{1}{N}{\sum }_{i=1}^{N}{({{\boldsymbol{\phi }}}_{{\mathrm{true}}}-{{\boldsymbol{\phi }}}_{s}^{(i)})}^{2}}).$$

The reported NRMSE is the mean of the parameters.

UMAP^80^ provides a nonlinear embedding that preserves local and global relationships in complex data, allowing meaningful comparison even when trajectories differ in length and structure. To quantify the discrepancy between the simulated and observed data, we performed a UMAP projection into a 10-dimensional space and computed the cosine similarity between each simulated and observed cell:

$${d}_{\cos }({x}_{i},{x}_{j})=\frac{{x}_{i}\cdot {x}_{j}}{| | {x}_{i}| | \,| | {x}_{j}| | }.$$

All UMAP hyperparameters were fixed to standard settings, and we padded not-observed time points with − 1.

### Implementation

We used the FitMultiCell^81^ pipeline to load the model from Morpheus^16^ and infer the parameters. One simulation takes between 10 and 30 s. For the ABC algorithm, we chose a sequential Monte Carlo implementation in pyABC^82^, setting a population size of 1000 particles and using stopping conditions of a minimal acceptance rate of 0.01 or a maximum of 15 generations. The acceptance threshold *ϵ* in each generation was set adaptively by calculating it as the median of the distances from the last population, as it provides a data-driven criterion that does not require manual tuning. The acceptance threshold for the first generation is calculated in a pre-calibration round. We parallelized the simulations in each generation to maximize resource efficiency. If no cells were observed in the simulation, we set the Wasserstein distance to *∞*.

We built the NPE using BayesFlow^83^. Using neural spline flows as an inference network^84^, we trained networks with 6–8 layers. The smallest network served for inference, while all three together were employed as an ensemble network, i.e., samples of all networks were combined to obtain more conservative posterior estimates, as proposed by ref. ^39^, and to check for model misspecification^85,86^. Additionally, we regularized the summary latent space to be normally distributed such that we can check for model misspecification if the real data is far from the simulated data^43^.

To extract informative representations from time-resolved cellular data, we designed a summary network composed of convolutional, recurrent, and attention-based components. Each trajectory was first passed independently through a one-dimensional convolutional layer that captured local temporal features. This is followed by a gated recurrent unit (GRU) layer with 32 units, which models sequential dependencies across time points^87^. Both layers operate independently on each cell, ensuring parallel processing across cells. We implemented a temporal attention mechanism to aggregate the latent representations over time. A global query vector was computed by averaging the GRU outputs across all cells. This query is then used to attend to the time steps of each cell, enabling the model to focus on the most informative periods of each trajectory. The attended sequence is subsequently compressed via a pooling operation, resulting in a fixed-dimensional vector (dimension 8) that serves as a population-level summary statistic. Time points were appended to the coordinates, and missing observations in the trajectories (and observations outside the visible window in the experiment) were set to 0 and encoded by an additional binary indicator, as suggested by ref. ^88^. This resulted in a 4-dimensional tensor as input to the summary network. The standalone training of the summary network was performed by reducing the summary dimension to the number of parameters and minimizing the mean squared distance of the predictions and parameter values to approximate the posterior mean^32^.

We used the same training set for the joint training of the summary and inference networks as for the standalone summary network. We performed 32,000 simulations with 143 cells in each, taking 1.39 h, employed a batch size of 32, and set 50 as the maximum number of epochs. From a further 300 simulations, we built a validation set and employed an early stopping. All simulations and parameters were standardized by computing the mean and standard deviation of each coordinate and parameter in the validation set. We trained with the Adam optimizer, an initial learning rate of 5 × 10^−4^, and employed a cosine-decay schedule, as it is the default in BayesFlow. The training time for the joint training was 2.64 h and for the standalone summary network, 4.04 h.

We ran all analyses on a computing cluster using 6 CPU nodes for parallelization, each with 32 cores, and one GPU for training the neural networks. The computing cluster used an Intel Xeon Sapphire Rapids CPU with a core clock speed of up to 2.1 GHz and 60 GB of RAM. The neural network training was performed on a cluster node with an Nvidia A40 graphics card with 48 GB of VRAM.

## Supplementary information

Supplementary Information

## Data Availability

The underlying code and experimental data for this study are available and can be accessed in this repository: https://github.com/emune-dev/Cell-Migration-Complex-Environments.git. Additionally, a snapshot of code and data is saved at Zenodo with 10.5281/zenodo.16893454, and the MorpheusML model is also provided via this identifier: https://identifiers.org/morpheus/M6342.
