# Supplementary material for: Simulation-based inference of cell migration dynamics in complex spatial environments
Source: NPJ Syst Biol Appl. 2026 Jan 29;12:20. doi: 10.1038/s41540-026-00648-9 (PMC12873338; doi:10.1038/s41540-026-00648-9)
Supplement: Supplementary file 1 — Supplementary Information [file 41540_2026_648_MOESM1_ESM.pdf]

Supporting information

Additional results for the simulated dataset

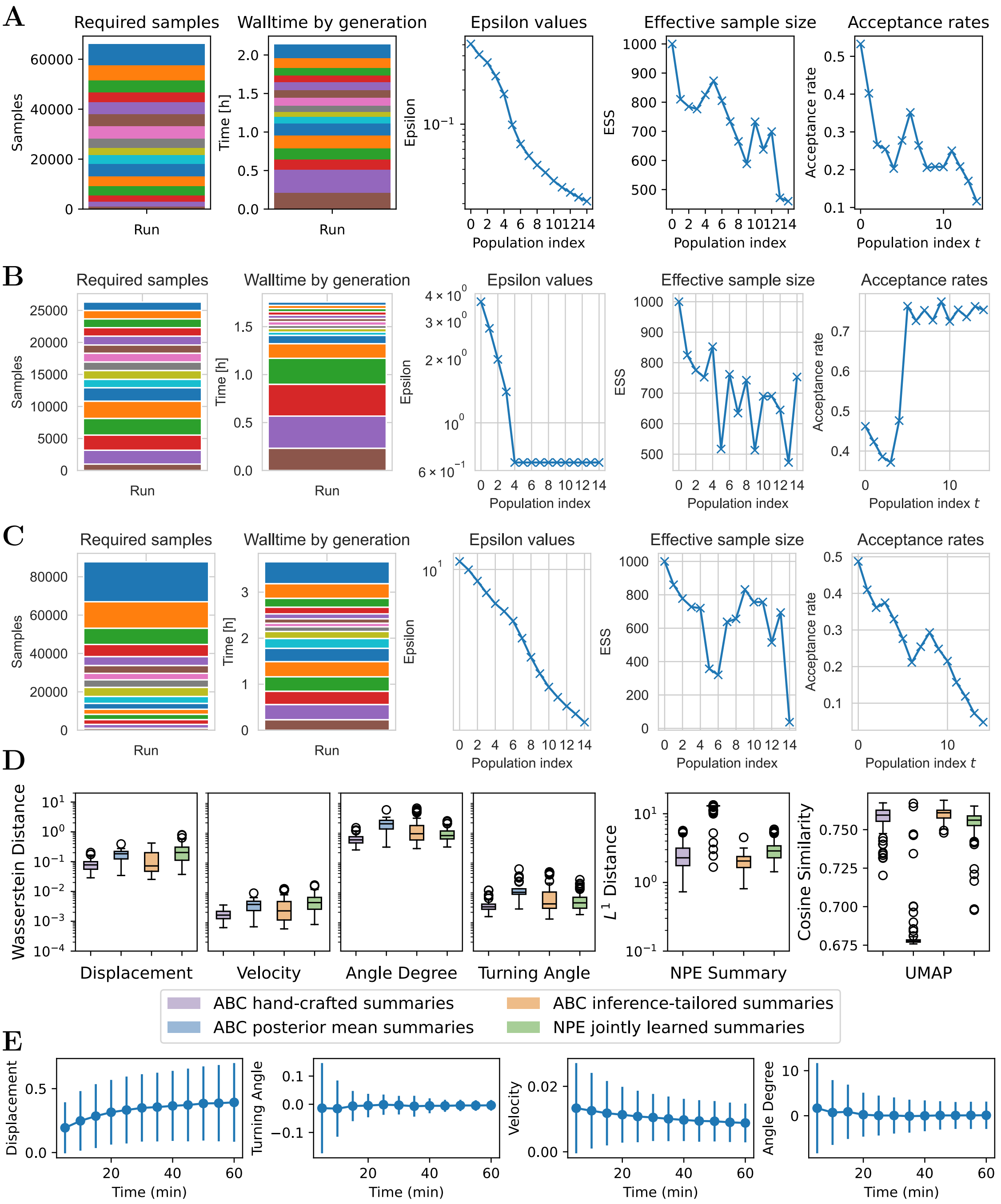

Supplementary Figure 1: *Diagnostics of ABC for each generation of the sampling run on synthetic data set 1.* (**A**) Diagnostics of ABC with hand-crafted summaries. (**B**) Diagnostics of ABC-PM with posterior mean summaries. (**C**) Diagnostics of ABC-NPE with inference-tailored summaries. (**D**) Summary statistics for ABC and NPE. (**E**) Summary statistics computed at 10 min intervals for the full trajectory of the test simulation. We show mean and standard deviation over cells.

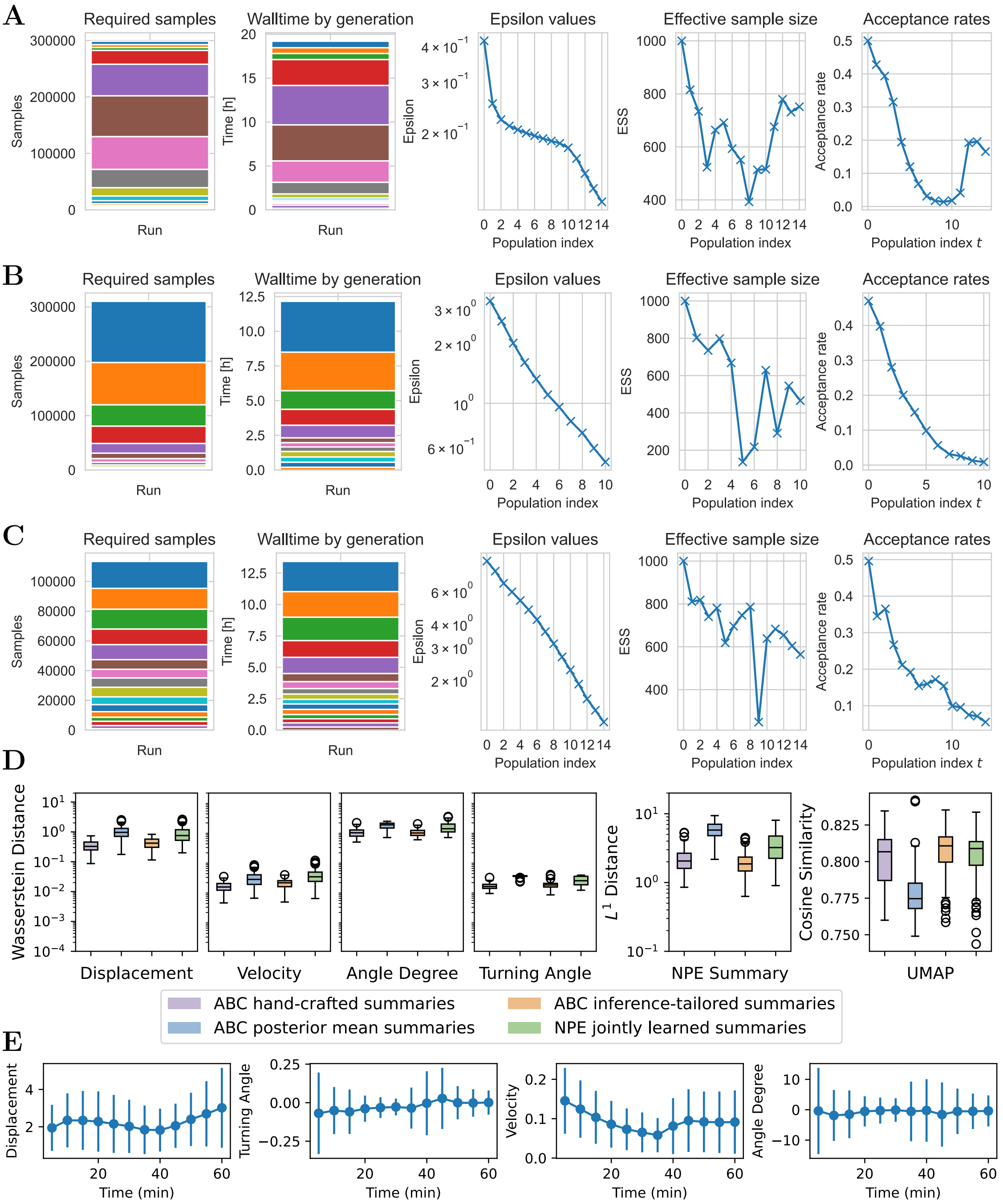

Supplementary Figure 2: *Diagnostics of ABC for each generation of the sampling run on synthetic data set 2.* (**A**) Diagnostics of ABC with hand-crafted summaries. (**B**) Diagnostics of ABC-PM with posterior mean summaries. (**C**) Diagnostics of ABC-NPE with inference-tailored summaries. (**D**) Summary statistics for ABC and NPE. (**E**) Summary statistics computed at 10 min intervals for the full trajectory of the test simulation. We show mean and standard deviation over cells.

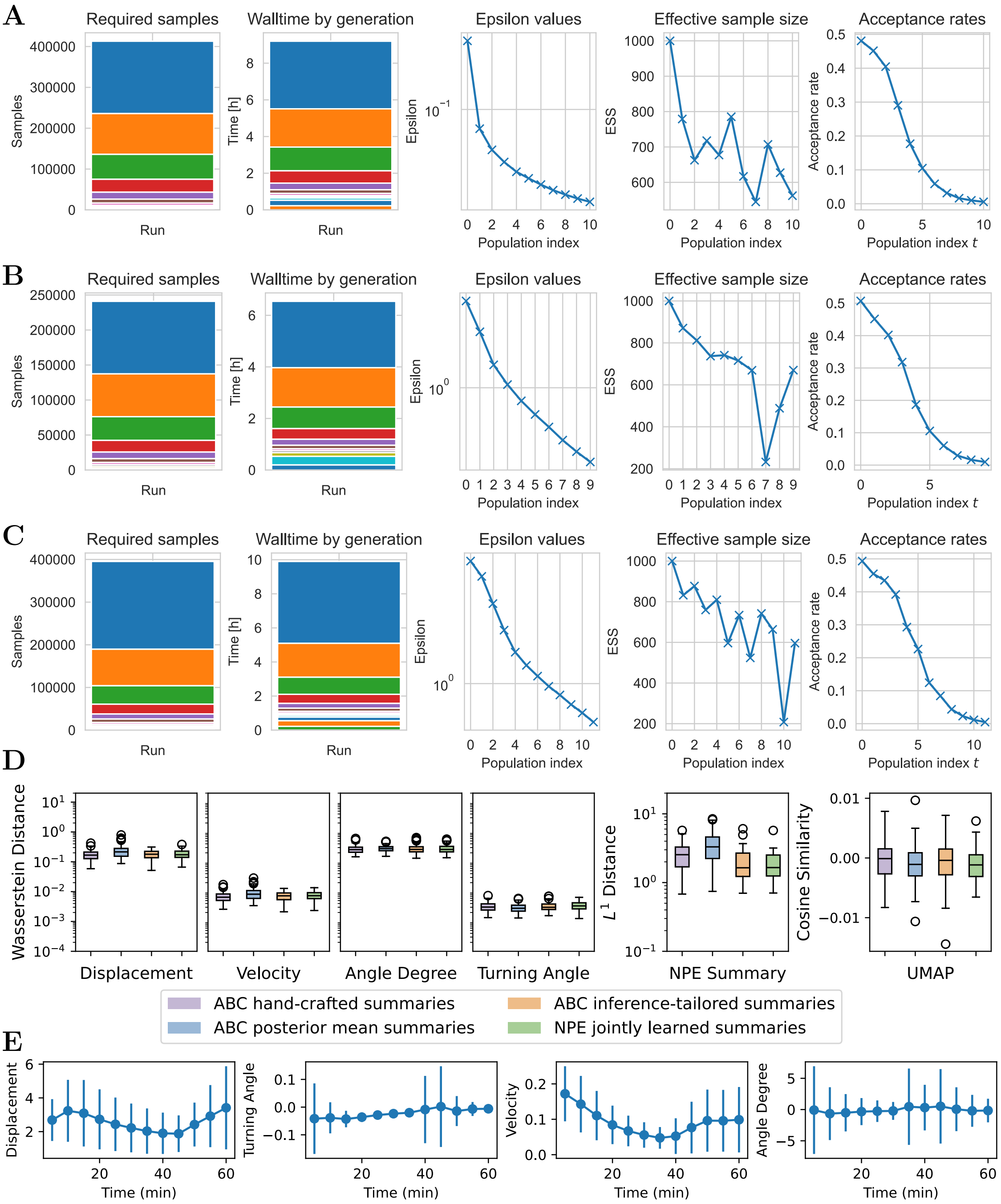

Supplementary Figure 3: *Diagnostics of ABC for each generation of the sampling run on synthetic data set 3.* (**A**) Diagnostics of ABC with hand-crafted summaries. (**B**) Diagnostics of ABC-PM with posterior mean summaries. (**C**) Diagnostics of ABC-NPE with inference-tailored summaries. (**D**) Summary statistics for ABC and NPE. (**E**) Summary statistics computed at 10 min intervals for the full trajectory of the test simulation. We show mean and standard deviation over cells.

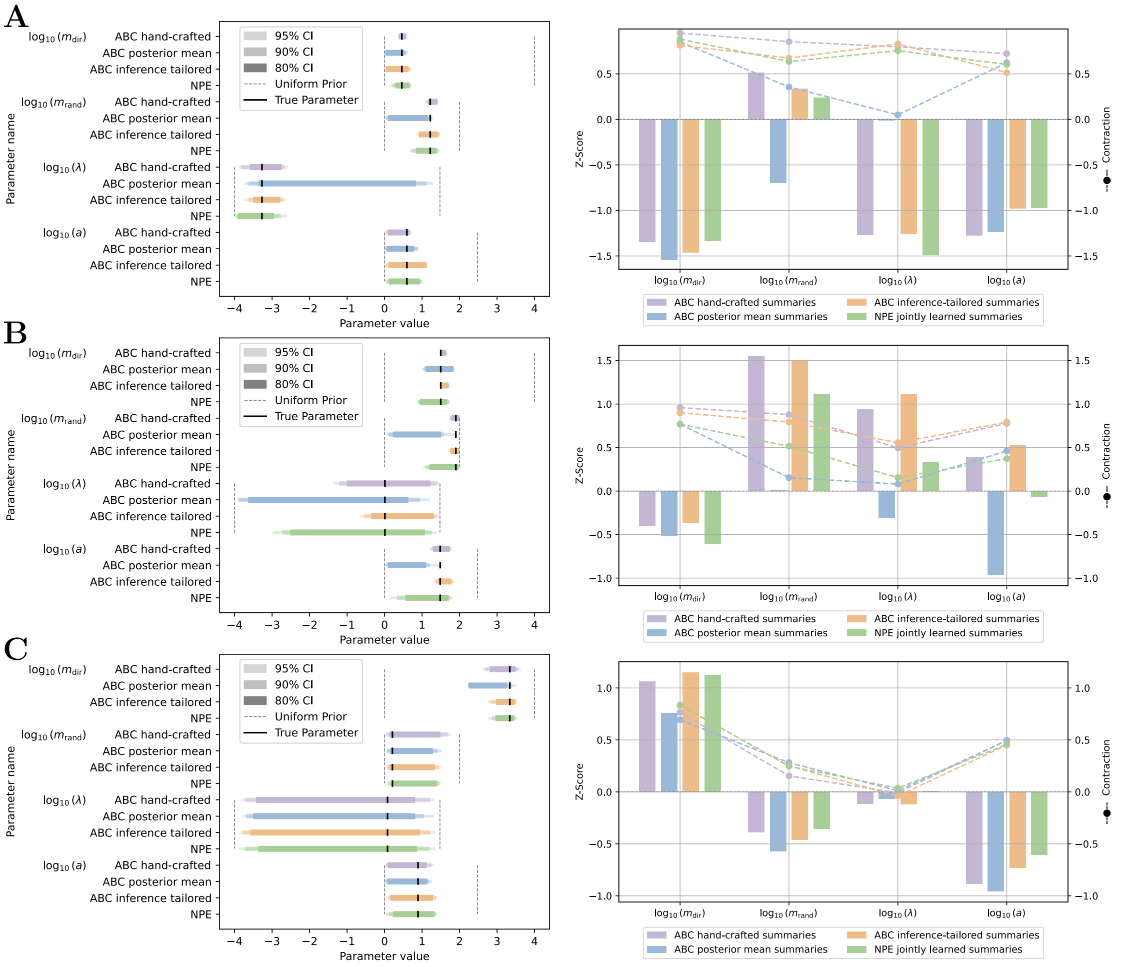

Supplementary Figure 4: *Credible intervals (left) and contraction (right) of the posteriors from the different approaches for the synthetic test data.* (**A**) Dataset 1. (**B**) Dataset 2. (**C**) Dataset 3.

Additional results for the experimental dataset

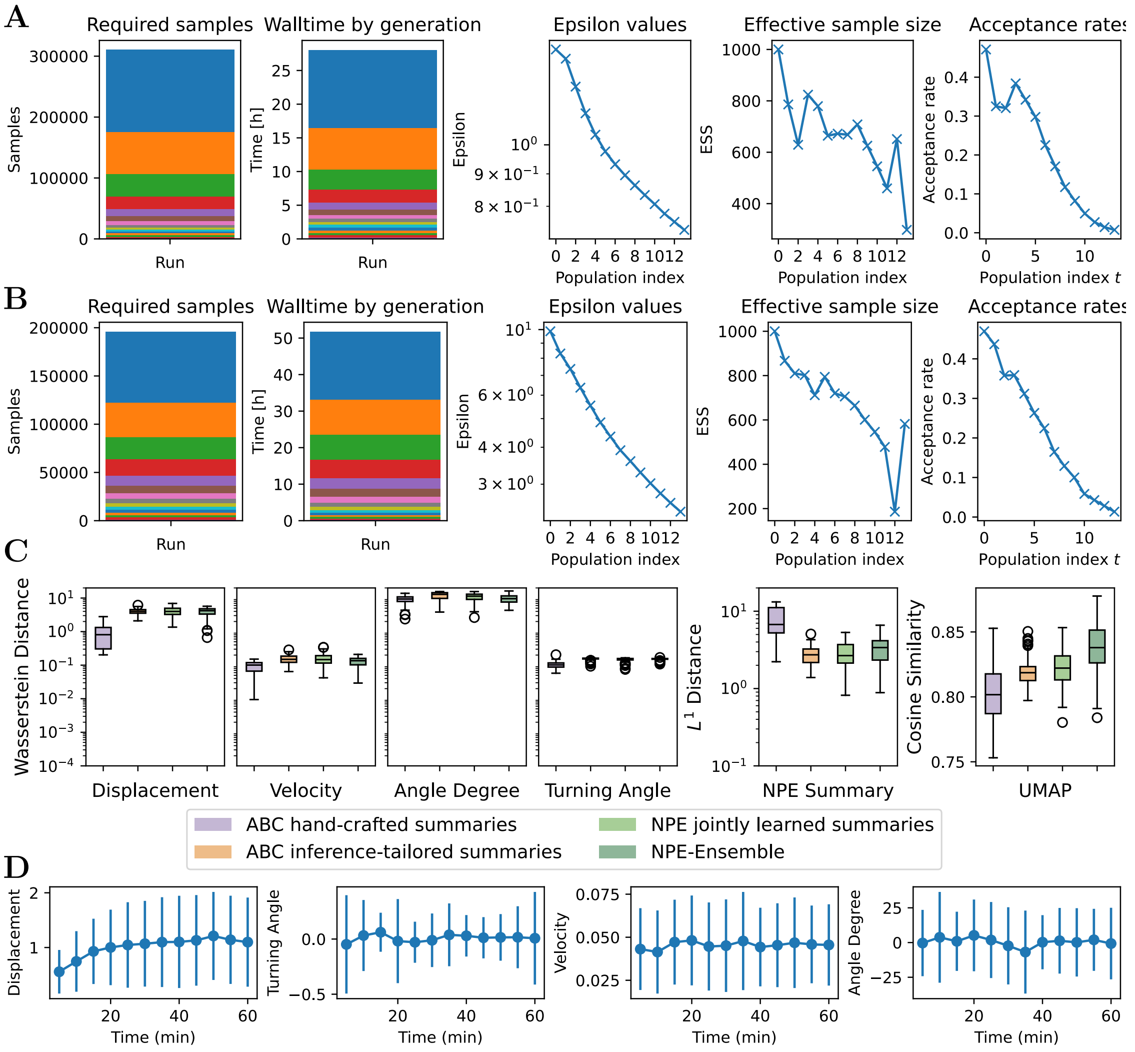

Supplementary Figure 5: *Diagnostics of ABC for each generation of the sampling run on the empirical data.* (**A**) Diagnostics for ABC with hand-crafted summaries on experimental data. (**B**) Diagnostics for ABC-NPE with learned summaries on experimental data. (**C**) Summary statistics for ABC and NPE. (**D**) Summary statistics computed at 10 min intervals for the full trajectory of the test simulation. We show mean and standard deviation over cells.

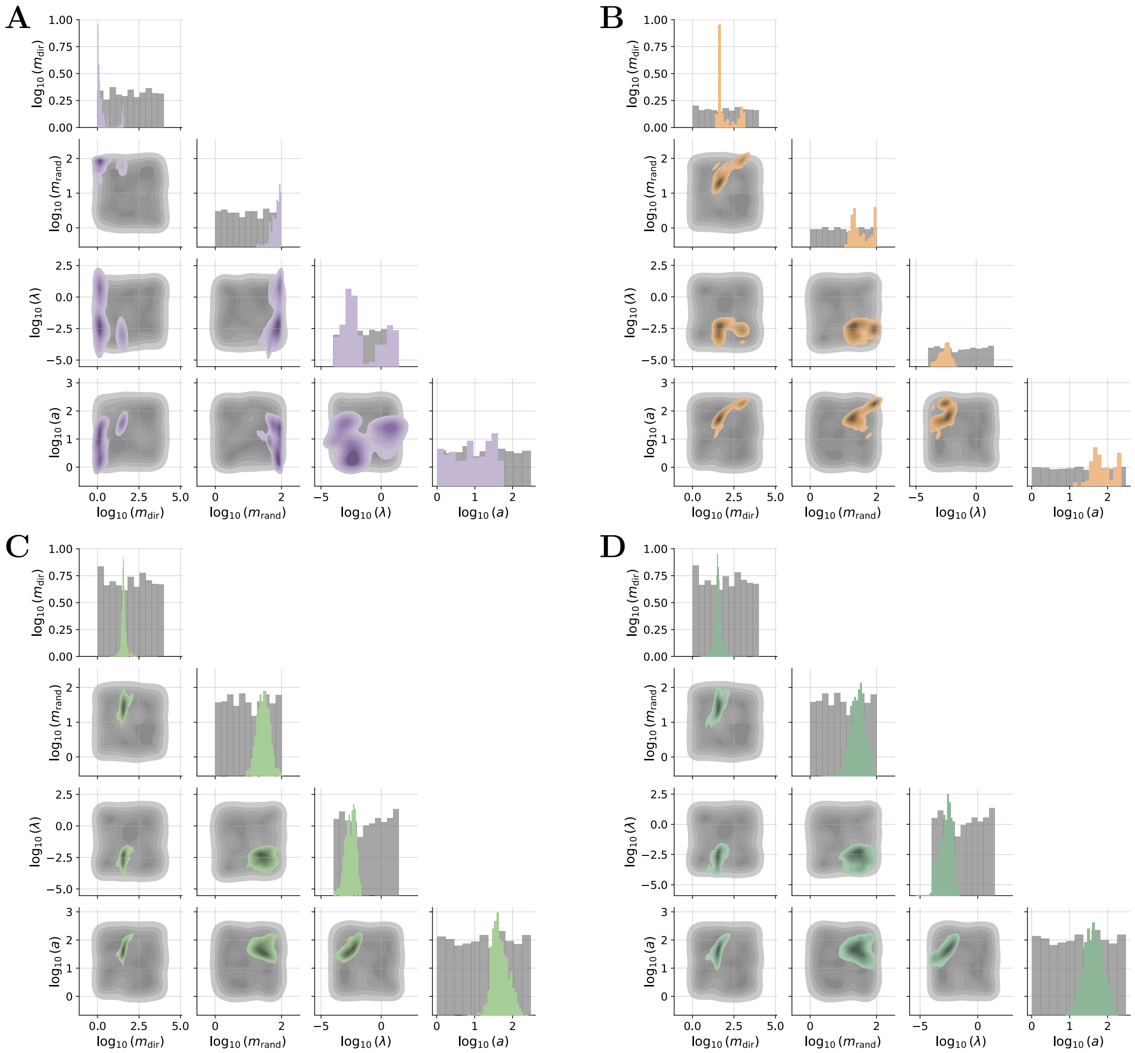

Supplementary Figure 6: *Posteriors for the emperical data.* The posterior is colored and prior is depicted in gray. (**A**) ABC with hand-crafted summaries. The smaller mode seems to be the key driver in increasing the uncertainty of the other parameters. (**B**) ABC-NPE m_{dir} shows a bimodality, but otherwise the posterior looks similar to the NPE approaches. (**C**) NPE shows not sign of bimodality. (**D**) NPE-Ensemble has a slightly more conservative posterior than NPE.

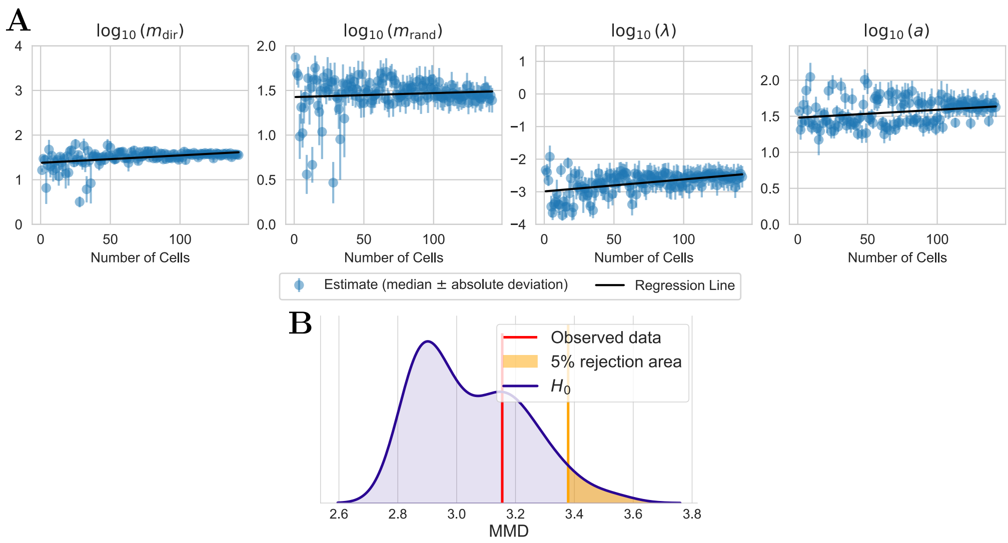

Supplementary Figure 7: *Additional robustness checks.* (**A**) Inference for increased number of cells in the experiment by randomly subsampling the dataset. (**B**) We can use the learned summary network to compare the summaries of the experimental data with those from the simulations observed during training. During training, we enforced the inlier summary distribution to be Gaussian. Then, misspecification can be detected by a distribution mismatch in the summary space with a high maximum mean discrepancy (MMD), as proposed by [42]. The reference distribution was constructed using the validation data. However, in this case, we cannot reject the hypothesis that the experimental data are consistent with the normal distribution, meaning that no misspecification was detected.

Additionally, we compute a matrix $K \in \mathbb{R}^{3\times 3}$ of pairwise Kullback–Leibler divergences between the posteriors inferred by each of the 3 models in the ensemble, as suggested by [85]. Each entry K_{ij} is estimated by averaging the log-density ratio between models i and j's posteriors over samples from model i. The magnitude of the entries in K serves as a diagnostic tool for ensemble consistency, with small values indicating similar predictive behavior. A heuristic condition for consistency is the normalized maximum divergence $\max_{i,j}(K_{ij}/d) \ll 1$, where d is the dimensionality of the parameter space. In our case, the maximum normalized value is 1.01 for 100 posterior samples, indicating rather high variability between the ensemble members, but no clear evidence of inconsistency within the ensemble.
